# NCAA Division I American football players with sickle cell trait have altered hematological responses and hydration status

**DOI:** 10.1038/s41598-021-81473-4

**Published:** 2021-01-19

**Authors:** Haoyan Wang, Matt Martone, Michael E. Owens, Nathan P. Lemoine, Jack Marucci, Derek Calvert, Shelly Mullenix, Timothy S. Church, Jennifer Rood, Brian Harrell, Brian A. Irving, Guillaume Spielmann, Neil M. Johannsen

**Affiliations:** 1 College of Physical Education and Health Sciences, Zhejiang Normal University, Jinhua, 321000 China; 2 Department of Kinesiology, School of Kinesiology, Louisiana State University, Baton Rouge, LA 70803 USA; 3 Department of Athletics, Louisiana State University, Baton Rouge, LA 70803 USA; 4 Pennington Biomedical Research Center, Baton Rouge, LA 70808 USA; 5 Baton Rouge General, Baton Rouge, LA 70806 USA

**Keywords:** Biomarkers, Diseases, Health care, Risk factors

## Abstract

Sickle cell trait (SCT) is a risk factor of collapse and sudden death in athletes. We conducted a longitudinal study to determine the hematological responses and hydration status in NCAA Division I American football players with SCT. The study took place over 2 years with 6 SCT and 6 position-matched controls (CON) in year 1; and 4 SCT and 4 CON in year 2. In year 2, three of the four SCT players were recruited and re-enrolled with new position-matched controls (total sample data = 10 SCT and 10 CON). Blood samples were taken at three visits: pre-camp, post-camp, and post-season to examine hemoglobin variants, complete blood counts, and chemistry panel 26. Hydration status was assessed by measuring body weight change, urine specific gravity, and urine and sweat electrolyte concentrations during the pre-season training camp. All SCT players were confirmed to have SCT (HbS = 37.9 ± 2.4%) and had greater red cell distribution width (RDW) compared to CON across all visits. Serum uric acid was higher in SCT (7.3 ± 1.0 mg/dL) compared to CON (6.1 ± 0.6 mg/dL; p = 0.001). Furthermore, serum creatine kinase levels were greater in SCT (1617.0 ± 1034.8 IU/L) at pre-camp compared to CON (1037.4 ± 602.8 IU/L; p = 0.03). SCT players exhibited lower pre- and post-practice urine electrolytes and urine specific gravity (SCT pre: 1.019 ± 0.005 vs. CON pre: 1.026 ± 0.008 p < 0.001; SCT post: 1.020 ± 0.005 vs. CON post: 1.030 ± 0.008 p < 0.01), whereas sweat sodium concentrations were higher in SCT players (55.4 ± 13.6 mmol/L) compared to CON (45.5 ± 10.6 mmol/L; p < 0.001). Given the evidence, greater uric acid and CPK levels in SCT players compared to CON may be an early indicator of altered kidney function and muscle damage, which could be added into NCAA guidelines for surveillance among SCT players. Consistent education and reinforcement of the importance of adequate fluid balance during exercise are critical for both SCT and CON players.

## Introduction

Sickle cell trait (SCT) is a genetic condition characterized by the presence of hemoglobin S (HbS), which makes up approximately 40% of total hemoglobin^1^. The molecular structure of HbS reduces its oxygen affinity, resulting in less oxygen delivery to active muscle and contributing to local hypoxia, which may impact optimal performance in athletes^1,2^. SCT is seen in 7–9% of African Americans and up to 40% in Western Africans^3^. Previous studies have investigated the effects of acute exercise on SCT individuals, suggesting SCT carriers had greater blood viscosity and red blood cell rigidity at rest, during intensive training, and during the subsequent recovery^4^. In addition, a previous study demonstrated that greater blood viscosity should be considered a risk factor associated with exercise-related sudden death in SCT carriers^5^. The physiological and environmental stress during exercise including exercise-induced acidosis, dehydration, heat stress, and regional hypoxemia, promote hematological changes that can increase the risk of microcirculatory disorders, cardiovascular complications, and fatality in SCT carriers^4^.

American football players with SCT have an increased risk of complications due to the exhaustive nature of practice and competitions^6–8^. In addition, geographical location, specifically hot and humid environmental climates, provides an extra challenge to the players with SCT^9^. Previous reports showed that NCAA Division I football players with SCT experience a higher percentage of heat-related illness and exertional-related death, up to 16 to 21-fold compared to non-SCT players^2,10^. From 2000 to 2010, 16 deaths have occurred during sport-related conditioning and games in Division I football players, among which 10 (63%) were SCT carriers^10^. Hematological alterations, inflammation, and vascular adhesion have been proposed as potential mediators of the microcirculatory changes that induce sickle cell anemia^11,12^. Although a clear relationship exists between SCT and the risk of exertional-related sudden death in American collegiate football, the pathophysiology of sickling-induced hematological alterations is still unknown^8,13^. According to the NCAA guidelines, the primary prevention strategies of sickling crisis is includes (1) requiring institutions to confirm SCT status before enrollment in any athletic team and (2) continued education of SCT players to avoid heat stress and dehydration^7,8^. In SCT, acclimatization should be implemented slowly and gradually, allowing players to have ample rest intervals during exercise due to the nature of the deoxygenated form of HbS, and possibly resulting in greater muscle fatigue^4,14^. In addition, athletic training staff is trained to identify the signs and symptoms of sickle cell crisis and differentiate it from heat cramps^8,9^. To our knowledge, no study has investigated the effects of training and competitions across the entire season on SCT players. Therefore, the purpose of this study was to conduct a longitudinal study examining hematological responses and hydration status in NCAA Division I football players with SCT. We hypothesized that SCT players would exhibit altered hematological responses assessed periodically over an entire season. In addition, we hypothesized that, due to the NCAA guidelines, SCT players would have improved hydration status.

## Methods

### Participants

Twenty NCAA Division I football players were recruited in two consecutive years from a university located in the southeast United States. Players with SCT were approached by athletic training staff and 6 agreed to enroll in year 1. Four SCT players agreed to participate in year 2, of which, three of them were re-enrolled from year 1. In addition, 6 position-matched controls were enrolled during year 1 and 4 new CON players were enrolled in year 2 for a total of 10 SCT and 10 CON participants enrolled in this study. The CON participants were also selected by the athletic training staff to have similar anthropometric characteristics with similar training repetitions and drills compared to their SCT counterparts. SCT was confirmed by the hemoglobin characterized by the presence of HbS, but less than 50%, and a normal percentage of HbA2^4,7^. The physical characteristics of the players are displayed in Table 1. The study was conducted according to relevant guidelines and regulations (Declaration of Helsinki) and the protocol and informed consent were approved by the Louisiana State University Institutional Review Board (IRB #3900). All participants signed informed consents before any assessments were completed.

**Table 1:** Physical characteristics of participants by groups.

	SCT (n = 10)	CON (n = 10)	*p* value
Age (year)	19.8 ± 1.6 (18–23)	21.1 ± 1.2 (19–23)	0.10
Height (cm)	186.7 ± 5.4 (177.8–193.1)	190.9 ± 4.9 (180.3–198.1)	0.08
Weight (kg)	110.6 ± 28.4 (80.0–154.7)	109.9 ± 24.7 (84.5–145.2)	0.91
Hemoglobin-A (%)	57.3 ± 2.5 (55.4–64.0)^a^	95.2 ± 0.4 (94.8–95.8)	< 0.001
Hemoglobin-A2 (%)	3.5 ± 0.2 (3.3–3.7)^a^	2.9 ± 0.2 (2.5–3.1)	< 0.001
Hemoglobin-F (%)	0.4 ± 0.2 (0.1–0.8)	0.5 ± 0.2 (0.2–0.9)	0.48
Hemoglobin-S (%)	37.9 ± 2.4 (31.4–40.1)^a^	0	< 0.001
Hemoglobin-C (%)	0	0	N/A

Data are presented as mean ± SD (Range).

*SCT* sickle cell trait, *CON* position-matched control group.

^a^Significant difference between groups.

### Study design and visits

**Figure 1:**
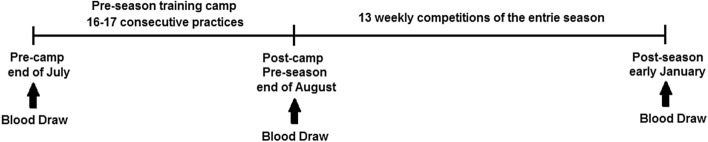
The time-point of the blood draws throughout the entire football season.

Blood samples were taken at three visits: (1) within a week prior to the start of training camp (pre-camp), (2) approximately 48 h after the end of pre-season training camp (post-camp/pre-season), and (3) approximately 48 h after the final competition of the season (post-season). There were 17 consecutive practices in the first year and 16 practices in the second year between pre-camp and post-camp and 13 weekly in-season competitions between post-camp and post-season (Fig. 1). Pre-camp data collection (blood samples) served as the baseline measurements. The follow-up measurements examined the alterations associated with practice and the competitive season. In addition, hydration status (urine and sweat) was examined at each practice in the training camp to determine group differences.

### Blood biomarkers

Venous blood was drawn from an antecubital vein in the non-fasted state, ~ 2–3 h post-prandial. Whole blood was collected in 7 mL (K2-EDTA) and 7 mL serum separator tubes, allowed to clot (serum tubes only), centrifuged for 10 min at 2000*g*, and the resultant plasma and serum transferred to cryovials for future analysis. Serum was analyzed for a comprehensive chemistry panel 26 (CM26). Additional whole blood samples (EDTA) were analyzed by electrophoresis to determine hemoglobin subtyping as well as complete blood cell counts (CBC). Hemoglobin electrophoresis subtypes included the percentage of hemoglobin-A, hemoglobin-A2, hemoglobin-F, hemoglobin-S, and hemoglobin C to diagnose sickle cell. In addition, the estimated glomerular filtration rate (eGFR) was calculated based on serum creatinine^15^.

### Measures of hydration status

Environmental conditions were measured before and after practice using a wet-bulb globe temperature (WBGT8758 Vernon Hills, IL USA). Body weight was assessed using a standard scale (TANITA, Arlington Heights, IL, USA) with a precision of 0.1 kg before and after each practice session. Players were asked to wipe down the body and completely empty their bladder before body weight assessments. In addition, the same amount of clothing was required before and after practice body weight assessments. Hydration status was represented as % body weight change (after toweling dry and urinating), accounting for fluid intake and urine output. Urine samples were collected before and after each practice session before body weight assessments to determine urine specific gravity (USG) and electrolyte concentrations. USG was measured using a spectral refractometer (ATAGO CO., Bellevue, WA, USA) and urine electrolyte concentrations (sodium [Na^+^], potassium [K^+^], and chloride [Cl^−^]) were measured by ion-selected probes (MEDICA EasyLyte; Bedford, MA, USA). In addition, sweat was collected on the lower back region using the technical absorbent patch technique^16^, which has been showed a valid and simple method to assess large number of players during on-field team sports^17^. The skin surface was cleaned with 70% ethanol and dried before the sweat patch was attached. Sweat patches were removed 60 min into each practice to avoid patch over-saturation. The local sweat rate was calculated as the weight differences of the absorbent patch pre- and post-practice divided by the product of the sweat patch area and duration of time the patch attached (mg/cm^2^/min). Sweat was analyzed for electrolyte concentrations (sodium [Na^+^], potassium [K^+^], and chloride [Cl^−^]) similarly to the urine. The sweat electrolyte concentrations and local sweat rate were corrected by the Baker et al. representing the whole body losses because urine volume and fluid intake were not feasible to assess without interrupting players’ training routine^18^.

### Statistical analysis

**Figure 2:**
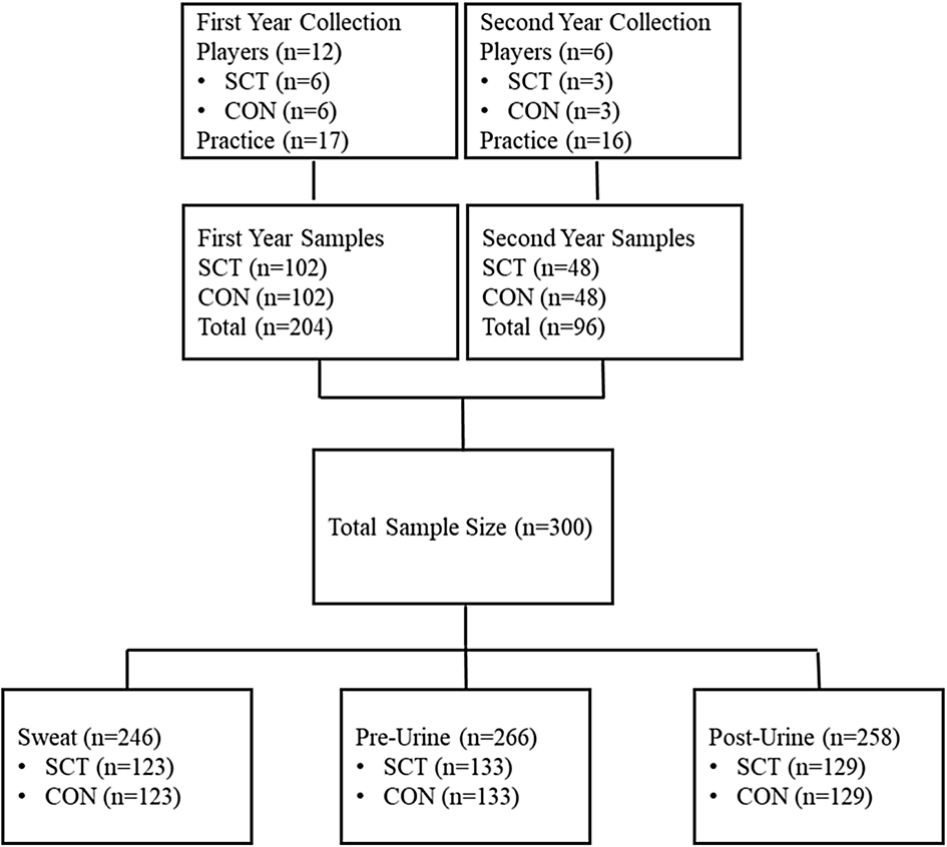
The total hydration samples were collected and pooled across practices by groups.

Data were analyzed using JMPro 14 (SAS Inc., Cary, NC). Given the participants were matched a prior based on their anthropometric, demographic, and on-field characteristics, the SCT and CON groups were analyzed using a within group structure as reported in this manuscript. However, all data were analyzed as if the SCT and CON groups were independent, with no major differences in the overall interpretation of the data. Baseline anthropometric and hematological outcomes were analyzed using a paired t-test. Two-way repeated-measures analysis of variance (RM ANOVA) was used to analyze hemoglobin electrophoresis, CBC, and CM26 between the two groups (SCT and CON) across assessment time (pre-camp, post-camp, post-season). In addition, a paired t-test was used to analyze outcome variables of hydration status between two groups (SCT and CON), including body weight change, sweat rate, sweat and urine electrolyte concentrations, and USG. All players participated in the blood draw across three visits, except 1 SCT and 1 CON missed the post-season visit in year 2. The total sample size for the hydration assessments is displayed in the Fig. 2. A total 18 players participated in training camp practice assessment as researchers did not have reasonable access to another two players from year 2 (1 SCT and 1 CON). They are not the same players who missed blood draw at post-season. Thirty-four samples were missed because of injury during practices. Twenty sweat samples were eliminated due to sweat patch dropped or sample contamination and 8 post-practice urine samples were missed because players left early for treatment. Missing data were eliminated and data were only entered in the final analysis if both the SCT and CON participated in training on any given day. Data are presented as mean ± standard deviation (SD) and statistical significance was accepted at p < 0.05.

## Results

Table 1 shows the SCT and CON participant characteristics. Age, weight, height, and hemoglobin F were not different between the in the SCT and CON groups. However, the groups differed by hemoglobin S fraction confirming the participants in the SCT were indeed SCT. In addition, the players were matched for race (all African American) and on-field position.

### Blood biomarkers

**Figure 3:**
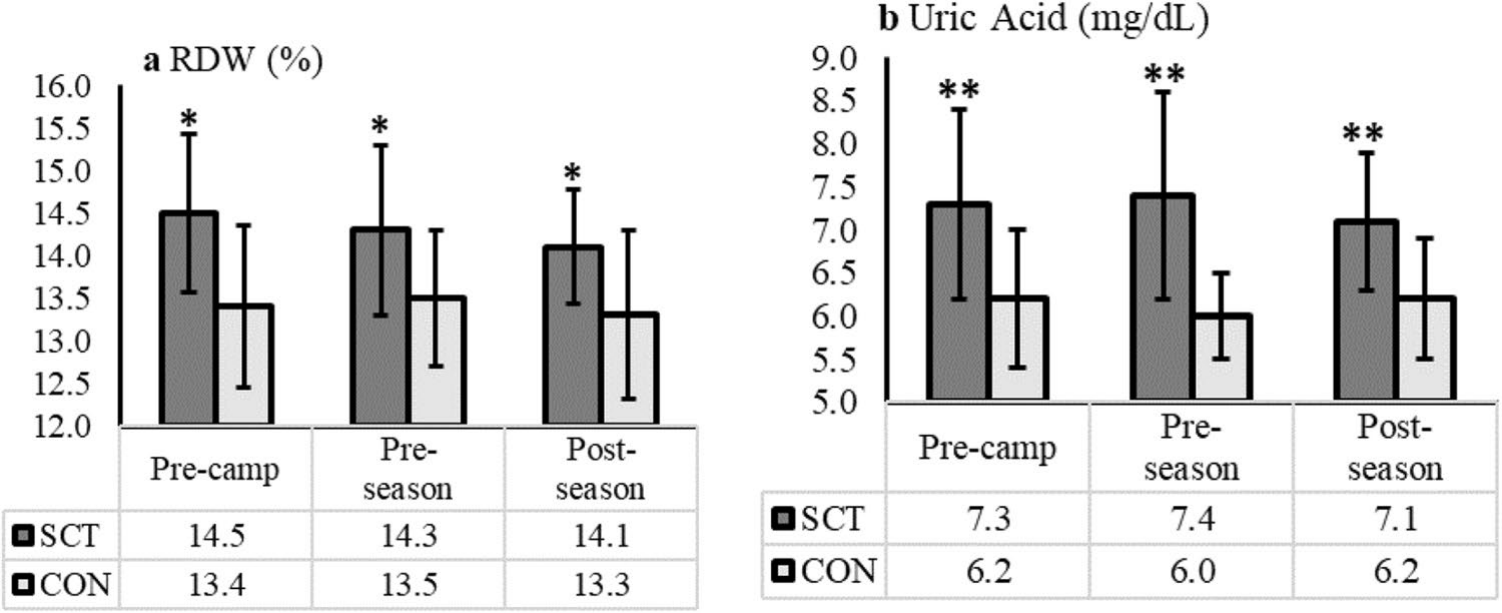
The red blood cell distribution width (RDW; **a**) and uric acid (**b**) across the visits by groups (SCT and CON). Dark grey bars are sickle cell trait players (SCT), and light grey bars are position-matched control group (CON). SCT players exhibited significantly greater RDW and uric acid across all visits compared to CON; *Significant differences p < 0.05. **Significant differences p < 0.01.

Hemoglobin-electrophoresis showed that SCT players had an average of 57.3 ± 2.5% HbA and 37.9 ± 2.4% HbS, and the percentages of HbA and HbS were consistent throughout the entire season (Table 1). The CBC profile is displayed in Table 2. A lower number of white blood cells (WBC; SCT: 5.6 ± 1.6 vs. CON: 6.9 ± 2.2 × 10^3; p = 0.02) and lymphocytes (SCT: 1.8 ± 0.5 vs. CON: 2.3 ± 0.9 × 10^3; p = 0.01) were observed in SCT compared to CON, but all data were within the normal reference range. In addition, SCT had a greater red cell distribution width (RDW) compared to CON (SCT: 14.3 ± 0.9 vs. CON: 13.4 ± 0.9%; p = 0.02) across all visits (Fig. 3a). Platelet count was significantly lower in SCT (197.9 ± 48.0 × 10^3) compared to CON only at post-season (278.7 ± 78.1 × 10^3; p = 0.03).

**Table 2:** The Completed Blood Count file of two groups (SCT vs. CON) across the entire football season (pre-camp, post-camp, and post-season).

Biomarkers (reference)	SCT (n = 10)				CON (n = 10)			
	Pre-camp	Post-camp	Post-season	Average	Pre-camp	Post-camp	Post-season	Average
RBC (4.5–5.9 × 10^6^)	5.1 ± 0.2	5.2 ± 0.2	5.3 ± 0.2	5.2 ± 0.2	4.9 ± 0.6	5.0 ± 0.6	5.1 ± 0.7	5.0 ± 0.6
Hb (14–18 g/dL)	14.1 ± 0.6	14.5 ± 0.8	14.7 ± 0.4	14.4 ± 0.7	14.1 ± 0.7	14.4 ± 0.7	14.8 ± 1.0	14.4 ± 0.8
Hematocrit (42–52%)	41.5 ± 1.8	42.3 ± 2.4	43.7 ± 1.2	42.4 ± 2.0	41.4 ± 1.9	42.0 ± 1.5	43.9 ± 2.5	42.4 ± 2.2
MCV (80–94 fL)	81.8 ± 2.7	81.4 ± 3.1	82.6 ± 3.1	81.9 ± 2.9	86.3 ± 8.8	85.6 ± 8.4	86.7 ± 9.1	86.2 ± 8.5
MCH (27–31 pg)	27.8 ± 1.3	27.9 ± 1.3	27.7 ± 1.2	27.8 ± 1.2	29.4 ± 3.4	29.5 ± 3.4	29.3 ± 3.5	29.4 ± 3.3
MCHC (32–36 g/dL)	34.0 ± 0.8	34.3 ± 0.5	33.6 ± 0.5	34.0 ± 0.7	34.1 ± 0.6	34.3 ± 1.0	33.7 ± 0.8	34.1 ± 0.8
RDW (11.5–14.5%)	14.5 ± 0.9^b^	14.3 ± 1.0^c^	14.1 ± 0.7^d^	14.3 ± 0.9^a^	13.5 ± 1.0	13.5 ± 0.8	13.3 ± 1.0	13.4 ± 0.9
Platelet count (150–450 × 10^3^)	205.9 ± 41.3	217.1 ± 60.9	197.9 ± 48.0^d^	207.2 ± 49.5	246.3 ± 58.7	251.0 ± 60.5	278.7 ± 78.1	256.0 ± 65.1
WBC (3.6–8.9 × 10^3^)	6.1 ± 1.9	5.7 ± 1.8	5.1 ± 1.0	5.6 ± 1.6^a^	7.8 ± 2.7	5.8 ± 1.0	7.0 ± 2.2	6.9 ± 2.2
Neutrophil (42.2–75.2%)	53.7 ± 6.2	58.3 ± 14.7	48.7 ± 13.5	53.7 ± 12.2	56.5 ± 13.1	55.2 ± 10.8	48.6 ± 12.7	53.6 ± 12.3
Lymphocyte (20.5–51.1%)	33.1 ± 6.1	30.6 ± 10.9	34.4 ± 6.8	32.6 ± 8.1	32.2 ± 10.9	34.4 ± 10.6	39.6 ± 11.6	35.2 ± 11.0
Monocytes (1.7–9.3%)	8.9 ± 3.1	8.2 ± 2.7	10.9 ± 3.1	9.3 ± 3.1	8.9 ± 3.3	7.7 ± 1.0	8.7 ± 3.2	8.4 ± 2.7
Eosinophils (0–10%)	5.1 ± 5.0^b^	2.3 ± 2.9	5.0 ± 5.9	4.1 ± 4.8	1.7 ± 1.5	2.2 ± 1.2	2.5 ± 1.8	2.1 ± 1.5
Basophils (0–0.8%)	0.6 ± 0.2	0.7 ± 0.5	1.0 ± 0.5	0.8 ± 0.4	0.6 ± 0.1	0.6 ± 0.3	0.8 ± 0.2	0.7 ± 0.2
Num of neutrophil (1.5–5.9 × 10^3^)	3.3 ± 1.1	3.5 ± 1.8	2.5 ± 0.9	3.1 ± 1.3	4.7 ± 2.9	3.3 ± 1.1	3.4 ± 1.4	3.8 ± 2.0
Num of lymphocyte (1.2–3.5 × 10^3^)	2.0 ± 0.7	1.6 ± 0.4	1.7 ± 0.4^d^	1.8 ± 0.5^a^	2.3 ± 0.6	2.0 ± 0.5	2.8 ± 1.4	2.3 ± 0.9
Num of monocytes (0.1–0.6 × 10^3^)	0.6 ± 0.3	0.4 ± 0.1	0.5 ± 0.2	0.5 ± 0.2	0.7 ± 0.2	0.4 ± 0.1	0.6 ± 0.2	0.6 ± 0.2
Num of eosinophils (0–0.7 × 10^3^)	0.2 ± 0.3	0.1 ± 0.1	0.2 ± 0.3	0.2 ± 0.2	0.1 ± 0.1	0.1 ± 0.1	0.2 ± 0.1	0.2 ± 0.1
Num of basophils (0–0.2 × 10^3)	0.02 ± 0.04	0.02 ± 0.04	0.03 ± 0.05	0.02 ± 0.04	0.02 ± 0.04	0.03 ± 0.05	0.07 ± 0.05	0.04 ± 0.05
Reticulocytes (0.9–2.3%)	1.4 ± 0.6	1.3 ± 0.4	1.2 ± 0.5	1.3 ± 0.5	1.3 ± 0.5	1.4 ± 0.6	1.2 ± 0.5	1.3 ± 0.5

Data presented as mean ± SD.

*SCT* sickle cell trait, *RBC* red blood cells, *Hb* hemoglobin, *MCV* corpuscular volume, *MCH* corpuscular volume, *MCHC* corpuscular hemoglobin concentration, *RDW* red cell distribution width, *WBC* white blood cell count.

^a^Significant difference in SCT compared to CON.

^b^Significant difference in SCT compared to CON at pre-camp.

^c^Significant differences in SCT compared to CON at pre-season/post-camp.

^d^Significant difference in SCT compared to CON at post-season.

**Figure 4:**
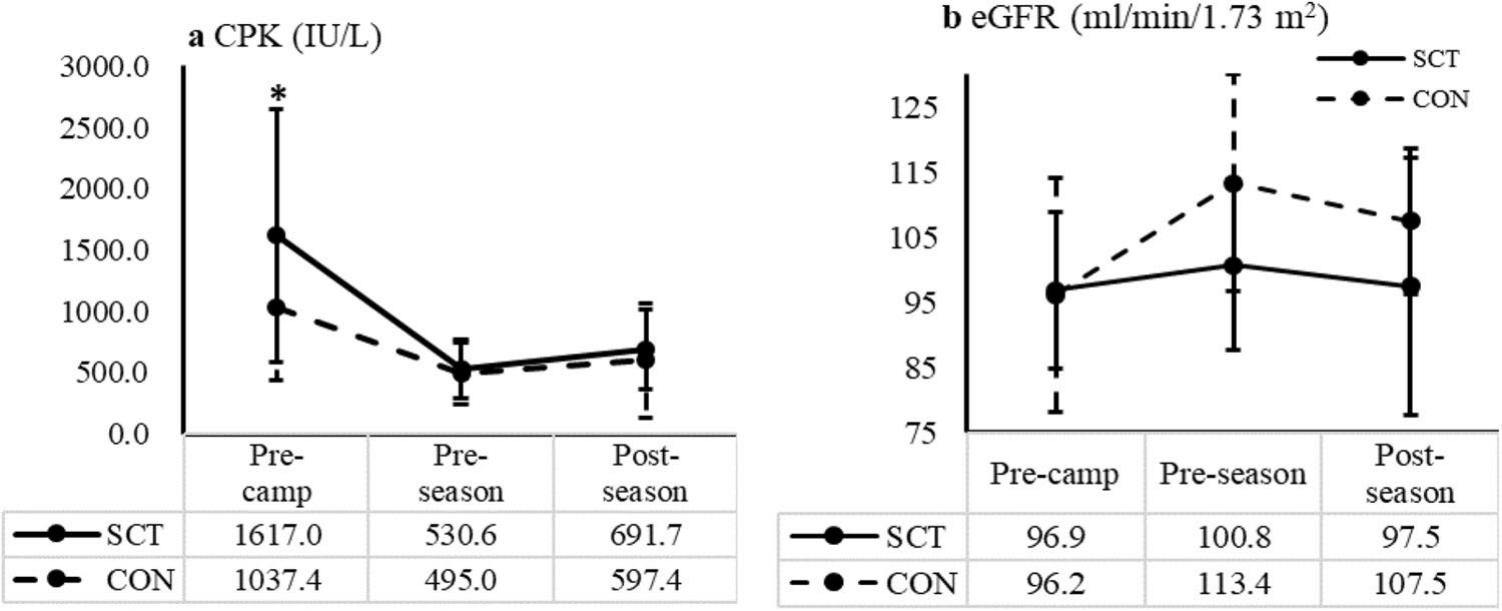
The CPK (creatine phosphokinase; **a**) and eGFR (estimated glomerular filtration rate; **b**) across the visits by groups (SCT and CON). Solid line indicates sickle cell trait players (SCT), and dash line indicates position-matched control group (CON). CPK levels were found significantly greater in SCT (1617.0 ± 1034.8 IU/L) at pre-camp compared to CON (1037.4 ± 602.8 IU/L; p = 0.04). The eGFR was lower in SCT at pre-season and post-season but not significant compared to CON (all p > 0.1). The eGFR was estimated by the equation from Shlipak et al.^15^.

Data from the CM26 can be found in Table 3. The BUN/Creatinine ratio was lower in SCT (12.5 ± 3.7) compared to CON at post-camp (17.0 ± 4.1; p = 0.003). Of note, SCT players had a greater uric acid and near to upper reference range (7.3 ± 1.0 mg/dL) compared to CON throughout the entire season (6.1 ± 0.6 mg/dL; p = 0.001; Fig. 3b). In addition, SCT players had greater CPK levels (955.2 ± 797.2 IU/L) compared to CON (718.1 ± 510.2 IU/L; p = 0.03), which was mainly observed at pre-season (SCT: 1617.0 ± 1034.8 vs. CON: 1037.4 ± 602.8 IU/L; p = 0.03; Fig. 4a). In addition, although no differences in LDH were found between two groups, both groups had elevated LDH that was greater than the upper reference range (SCT: 217.1 ± 34.1 IU/L and CON: 218.5 ± 44.7 IU/L; Reference 82 ~ 195 IU/L). Lastly, SCT players showed a lower eGFR at post-camp (pre-season) and post-season compared to CON, but these differences did not reach the level of statistical significance (all p > 0.1; Fig. 4b).

**Table 3:** Chemistry panel 26 profile of the two groups (SCT vs. CON) across the entire football season (pre-camp, post-camp, and post-season).

Biomarkers (reference)	SCT (n = 10)				CON (n = 10)			
	Pre-camp	Post-camp	Post-season	Average	Pre-camp	Post-camp	Post-season	Average
Glucose (70–110 mg/dL)	88.8 ± 13.4	85.3 ± 9.6	86.3 ± 14.4	86.8 ± 12.2	90.8 ± 21.2	86.1 ± 13.8	97.6 ± 32.2	90.7 ± 22.6
BUN (8–23 mg/dL)	17.5 ± 3.1	14.8 ± 3.2	14.8 ± 2.1	15.6 ± 3.1	19.4 ± 3.7	18.1 ± 3.0	15.0 ± 2.7	17.9 ± 3.5
Creatinine (0.9–1.3 mg/dL)	1.3 ± 0.1	1.2 ± 0.1	1.3 ± 0.2	1.2 ± 0.2	1.3 ± 0.2	1.1 ± 0.1	1.1 ± 0.1	1.2 ± 0.2
BUN/creatinine ratio (6–25)	14.0 ± 2.1	12.5 ± 3.7^c^	12.0 ± 2.8	12.9 ± 2.9	15.5 ± 2.4	17.0 ± 4.1	13.0 ± 2.5	14.9 ± 3.3
Sodium (136–147 mmol/L)	137.8 ± 1.2	137.3 ± 1.6	136.9 ± 1.1	137.3 ± 1.3	138.3 ± 1.2	137.1 ± 1.4	138.1 ± 1.0	137.9 ± 1.3
Chloride (101–111 mmol/L)	104.1 ± 0.7	103.8 ± 0.8	103.1 ± 2.2	103.7 ± 1.4	103.8 ± 1.8	103.8 ± 1.3	103.6 ± 1.2	103.6 ± 1.4
Potassium (3.7–5.0 mmol/L)	4.1 ± 0.2	4.5 ± 0.4	4.2 ± 0.5	4.3 ± 0.4	4.2 ± 0.3	4.5 ± 0.2	4.1 ± 0.3	4.3 ± 0.3
CO_2_ (25–33 mmol/L)	24.2 ± 1.4	25.1 ± 1.8	23.6 ± 2.1	24.3 ± 1.8	24.4 ± 2.2	25.4 ± 1.1	21.2 ± 5.3	24.1 ± 3.6
Uric Acid (2.6–7.5 mg/dL)	7.3 ± 1.1^b^	7.4 ± 1.2^c^	7.1 ± 0.8^d^	7.3 ± 1.0^a^	6.2 ± 0.8	5.9 ± 0.5	6.0 ± 0.4	6.1 ± 0.6
Total Protein (5.8–7.9 g/dL)	7.2 ± 0.3	7.3 ± 0.3	7.5 ± 0.1	7.3 ± 0.3	7.3 ± 0.4	7.4 ± 0.4	7.6 ± 0.3	7.4 ± 0.4
Albumin (4.1–5.4 g/dL)	4.4 ± 0.3	4.4 ± 0.3	4.4 ± 0.2	4.4 ± 0.3	4.4 ± 0.3	4.3 ± 0.3	4.4 ± 0.2	4.4 ± 0.3
Calcium (8.9–10.4 mg/dL)	9.3 ± 0.4	9.4 ± 0.3	9.4 ± 0.3	9.4 ± 0.3	9.4 ± 0.2	9.4 ± 0.2	9.5 ± 0.2	9.4 ± 0.2
Phosphorus (2.5–5.1 mg/dL)	4.2 ± 0.5	4.3 ± 0.6	4.1 ± 0.6	4.2 ± 0.5	4.3 ± 0.5	4.0 ± 0.4	4.1 ± 0.9	4.1 ± 0.6
Magnesium (1.7–2.4 mg/dL)	2.1 ± 0.1	2.1 ± 0.1	2.1 ± 0.1	2.1 ± 0.1	2.2 ± 0.1	2.1 ± 0.1	2.0 ± 0.1	2.1 ± 0.1
Total Bilirubin (0.2–1.5 mg/dL)	0.8 ± 0.3	0.9 ± 0.4	1.0 ± 0.2	0.9 ± 0.3	1.0 ± 0.4	1.1 ± 0.4	1.0 ± 0.4	1.0 ± 0.4
CPK (38–333 IU/L)	1617.0 ± 1034.8^b^	530.6 ± 236.5	691.6 ± 325.3	955.2 ± 797.2^a^	1037.4 ± 602.8	489.1 ± 268.0	451.8 ± 238.0	718.1 ± 510.2
LDH (82–195 IU/L)	207.1 ± 65.5	197.5 ± 82.5	186.7 ± 73.4	197.4 ± 72.1	217.1 ± 34.1	228.9 ± 37.7	225.8 ± 61.4	218.5 ± 44.7
AST (13–47 IU/L)	47.5 ± 21.4	31.6 ± 7.8	34.1 ± 8.6	37.9 ± 15.5	36.4 ± 10.4	32.2 ± 5.4	35.6 ± 11.7	35.0 ± 9.4
ALT (10–60 IU/L)	32.3 ± 12.6	26.7 ± 9.3	24.7 ± 10.2	28.0 ± 10.9	31.2 ± 10.9	25.6 ± 11.5	31.0 ± 14.9	29.2 ± 11.6
ALK Phosphate (32–130 IU/L)	88.5 ± 29.4	84.1 ± 22.7	83.1 ± 25.8	85.3 ± 25.3	74.0 ± 24.5	72.1 ± 23.2	77.5 ± 23.4	74.3 ± 21.7
GGT (5–63 IU/L)	20.9 ± 11.8	18.5 ± 11.9	16.4 ± 5.0	18.7 ± 10.0	17.7 ± 8.9	14.8 ± 12.3	19.0 ± 17.4	17.7 ± 12.2
Amylase (28–100 U/L)	116.9 ± 57.1	99.9 ± 37.5	99.6 ± 42.3	105.9 ± 45.7	91.1 ± 45.2	64.6 ± 7.7	67.9 ± 8.4	87.9 ± 48.4
Iron (50–160 μg/dL)	65.2 ± 24.7	86.0 ± 24.0^c^	100.6 ± 30.9	83.3 ± 29.5	73.1 ± 28.5	109.2 ± 34.1	81.1 ± 23.6	92.7 ± 38.3
Cholesterol (122–244 mg/dL)	166.2 ± 25.3	172.4 ± 26.7	174.1 ± 26.2	170.8 ± 25.3	160.7 ± 21.3	164.9 ± 10.1	166.3 ± 16.0	160.8 ± 17.7
Triglycerides (44–201 mg/dL)	91.0 ± 57.5	106.4 ± 63.3	67.6 ± 5.7	89.8 ± 51.9	64.2 ± 30.0	117.6 ± 75.4	82.6 ± 43.2	89.9 ± 55.1
HDL (30–70 mg/dL)	49.2 ± 12.6	54.4 ± 16.3	53.7 ± 12.9	52.4 ± 13.8	61.1 ± 9.5	58.7 ± 13.2	62.6 ± 12.2	61.3 ± 11.0
LDL (66–165 mg/dL)	98.8 ± 23.5	99.8 ± 19.2	108.5 ± 19.8	102.0 ± 20.7	86.7 ± 23.3	82.6 ± 9.7	87.1 ± 21.9	82.8 ± 19.4

Data presented as mean ± SD.

*BUN* blood urine nitrogen, *CPK* creatine kinase, *LDH* lactate dehydrogenase, *AST* aspartate amino transferase, *ALT* alanine amino transferase, *ALK* alkaline phosphate, *GGT* gamma-glutamyl transferase.

^a^Significant differences in SCT compared to CON.

^b^Significant difference in SCT compared to CON at pre-camp.

^c^Significant difference in SCT compared to CON at pre-season/post-camp.

^d^Significant difference in SCT compared to CON at post-season.

### Hydration status

Hydration markers derived from urine and sweat samples were collected during practices in the pre-season training camp under averaged 26.8 ± 1.9 °C (WBGT) at a relative humidity of 68.9 ± 11.1%. The hydration markers are presented in Table 4. CON experienced greater body weight loss (− 1.3 ± 1.1 kg; − 1.0 ± 0.9% body weight) and % body weight changes compared to SCT (− 0.9 ± 0.9 kg; − 0.8 ± 0.8% body weight; all p < 0.001). SCT players had lower pre- and post-practice urine electrolyte concentrations ([Na^+^], [K^+^], and [Cl^−^]) compared to CON. In addition, a lower pre- and post-practice USG was observed in SCT compared to CON (SCT pre: 1.019 ± 0.006 vs. CON pre: 1.026 ± 0.008, p < 0.001; SCT post: 1.020 ± 0.005 vs. CON post: 1.030 ± 0.008, p < 0.001). Sweat sodium concentrations (SCT: 55.4 ± 13.6 vs. CON: 45.5 ± 10.6 mmol/L; p < 0.001), potassium concentrations (SCT: 4.7 ± 1.2 vs. CON: 4.3 ± 0.9 mmol/L; p = 0.004), and chloride concentrations (SCT: 52.5 ± 13.0 vs. CON: 43.3 ± 9.9 mmol/L; p < 0.001) were greater in SCT compared to CON. However, sweat rate was not different between the two groups (SCT: 1.22 ± 0.22 vs. CON: 1.25 ± 0.25 mg/cm^2^/min; p = 0.32).

**Table 4:** Football pre-season camp practice hydration markers by groups.

Variables	SCT (n = 9)	CON (n = 9)	*p* value
Body weight loss (kg)	− 0.9 ± 0.9 (− 3.6 to − 1.8)^a^	− 1.3 ± 1.1 (− 4.5 to 1.8)	0.008
Body weight change (%)	− 0.8 ± 0.8 (− 3.5 to − 1.1)^a^	− 1.0 ± 0.9 (− 3.2 to 1.3)	0.04
Pre-urine Na^+^ (mmol/L)	98.4 ± 32.1 (19.4–167.6)^a^	159.6 ± 62.9 (27.9–284.1)	< 0.001
Pre-urine K^+^ (mmol/L)	37.2 ± 18.4 (7.4–115.4)^a^	55.8 ± 29.1 (3.8–152.2)	< 0.001
Pre-urine Cl^−^ (mmol/L)	88.5 ± 34.4 (14.4–177.2)^a^	150.2 ± 57.7 (24.2–290.9)	< 0.001
Pre USG	1.019 ± 0.005 (1.007–1.035)^a^	1.026 ± 0.008 (1.005–1.047)	< 0.001
Post-urine Na^+^ (mmol/L)	72.0 ± 27.2 (29.7–167.5)^a^	116.6 ± 52.5 (23.8–285.6)	< 0.001
Post-urine K^+^ (mmol/L)	37.4 ± 11.7 (11.7–80.8)^a^	63.8 ± 26.4 (14.5–169.9)	< 0.001
Post-urine Cl^−^ (mmol/L)	57.1 ± 28.1 (5.4–159.9)^a^	93.7 ± 48.6 (6.7–276.0)	< 0.001
Post USG	1.020 ± 0.005 (1.004–1.030)^a^	1.030 ± 0.008 (1.006–1.050)	< 0.001
Sweat Na^+^ (mmol/L)	55.4 ± 13.6 (29.6–88.6)^a^	45.5 ± 10.6 (25.1–74.5)	< 0.001
Sweat K^+^ (mmol/L)	4.7 ± 1.2 (3–7.2)^a^	4.3 ± 0.9 (2.9–6.8)	0.004
Sweat Cl^−^ (mmol/L)	52.5 ± 13.0 (25.9–83.4)^a^	43.3 ± 9.9 (23.3–71.9)	< 0.001
Sweat rate (mg/cm^2^/min)	1.22 ± 0.22 (0.74–1.82)	1.25 ± 0.25 (0.74–1.96)	0.32

*Pre* pre-practice, *Post* post-practice, *SCT* sickle cell players, *CON* position-matched control.

^a^Significant difference in SCT compared to CON, data were presented as mean ± SD (range) of averaged practices in the pre-season training camp.

## Discussion

The purpose of this study was to examine longitudinal changes in hematological biomarkers and hydration status in SCT players to better understand the training and seasonal implications and, based on our data, further develop prevention procedures based on NCAA sickling crisis precaution guidelines. Blood biomarkers revealed that SCT players had an average of 37.9% (31.4–40.1%) of HbS and greater RDW values than CON throughout the season. In addition, SCT players had higher serum uric acid throughout the entire season and higher CPK levels at pre-camp compared to CON. Training specific hydration markers showed SCT had an improved fluid balance compared to CON during pre-season training camp practice. In addition, SCT players had significantly lower pre- and post-practice urine electrolyte concentrations and USG, and higher sweat electrolyte concentrations compared to CON. Together, these data suggest that greater uric acid and CPK levels in SCT players compared to CON may be early indicated an altered kidney function and muscle damage. In addition, consistent reinforcement and educating the importance of adequate fluid balance during exercise are critical for both SCT and CON players.

The finding that SCT players had concomitant elevations in HbS and RDW is consistent with previous studies, that suggest HbS is associated with a higher RDW, which is related to the greater degree of red cell anisocytosis and larger variation of red blood cell morphology^19,20^. This finding further confirms the physiological alterations in red blood cells among asymptomatic SCT players. In the present study, the RDW value of SCT players was near the upper reference limit and previous research showed greater RDW correlated with the degree of anemia^19^. In addition, the immune cell counts appeared different between the two groups; however, the values remained in the normal reference range in both groups and do not appear to be clinically different. This evidence suggests that long-term conditioning and on-field practice do not affect immune function in SCT players. Given the difference noted in our study, additional studies may be necessary to delineate the clinical and physiological significance in the prevention of sickling crisis.

Surprisingly, serum uric acid remained higher throughout the season in SCT players compared to CON, suggesting either overproduction or insufficient clearance of uric acid. The potential mechanism proposed in the previous research is that serum uric acid is inversely associated with urine clearance rather than to the overproduction of uric acid^21^. Diamond et al. suggested that uric acid clearance was highly associated with renal excretion approaching the sum of new purine biosynthesis and dietary purine intake^22^. Previous studies have also reported that renal manifestations of an impaired urinary concentration are commonly observed in SCT carriers^23–25^. In our data, lower pre- and post-practice urine electrolyte concentrations in SCT players also suggest lower renal excretion to remove waste products through the urine. In addition, even though eGFR was not different between the two groups, SCT players showed a trend for having lower eGFR at post-camp (pre-season) and post-season. The eGFR is considered a good indicator of kidney function representing the ability of the kidneys to excrete fluid and waste product through urine^26^. We cannot conclude from our data that SCT players had an impaired kidney function or potential risk of kidney disease. Future studies could examine alternate measures of kidney function (e.g. urine creatinine), allowing for the direct assessment of urine clearance in SCT players.

Interestingly, a previous study reported that serum uric acid is an antioxidant that contributes > 50% of the total antioxidant capacity of the blood^27^. Thus, serum uric acid may play a protective role against exercise-induced oxidative stress^28^. In addition, previous studies showed that uric acid increased in response to exercise, such as short-distance running^29^, and ultramarathon racing^30^. The exercise-induced uric acid elevations may be associated with increased purine oxidation and subsequent formation of uric acid^31^. In the present study, our participants were long-term, highly trained elite collegiate American football players, and greater serum uric acid may be associated with the chronic exercise effects. To our knowledge, no studies have reported high serum uric acid in SCT players. However, the present study cannot explain whether greater serum uric acid levels observed in SCT players was associated with alterations of renal function or a protective mechanism related to reduced sickling activity. Future research could directly address this question, and thereby alter recommendations to athletic trainers and sport nutritionists to increase antioxidant intake if sickling can be prevented by the increase in serum antioxidant capacity.

Another important consideration is the effect of exercise on muscle damage in SCT players because previous studies frequently suggest SCT player are at a greater risk of exertional rhabdomyolysis^7,8,32^. Exertional rhabdomyolysis is a syndrome caused by the severe breakdown of skeletal muscle tissue leading to acute and chronic kidney failure^33^. Exertional rhabdomyolysis should be considered in NCAA football players with SCT, who are frequently exposed to demanding high-intensity exercise in hot and humid environmental conditions. In addition, laboratory tests have shown that serum CPK levels are elevated when rhabdomyolysis occurs^34^. The present study found that serum CPK levels were 3–5 times higher than the upper reference limits in both groups and at all visits. Furthermore, both groups had a significantly higher serum CPK levels at pre-camp compared to other visits. Moghadam-Kia et al. showed serum CPK levels could increase as much as 30 times of upper limit within 24 h of strenuous exercise^35^. A previous study also suggests that muscle mass and total body mass were associated with basal serum CPK levels^36^. In the present study, players’ average body weight was > 100 kg and greater serum CPK levels was possibly associated with this large body weight and muscle mass. In addition, both CON and SCT players had elevated LDH, indicating high degree of metabolic adaptation to the physical training of skeletal muscle^37^. Of note, serum CPK levels in the present study were greater at pre-camp in both groups and were significantly greater in SCT players. It is possible that the timing of the pre-camp blood draws coincided with heavy resistance training typical of American football players prior to the start of the season. As such, this increase in serum CPK levels prior to camp could be linked to methodological and sampling issues. On the other hand, this may suggest that SCT players had differential effects of exercise on muscle breakdown microvascular alterations, which can be the result of acute ischemia in skeletal muscle^38^. Future research should include comprehensive assessments of physiological work prior to all blood work to better elucidate the reasons for the elevated muscle damage markers in SCT.

Players with SCT are at greater risk of exercise-associated sickle cell collapse due to dehydration and heat stress^7,13^. In the present study, SCT players had an improved fluid balance, and both groups were hypohydrated with approximate − 1% body weight change and elevated USG throughout the training. In addition, greater pre-practice USG ≥ 1.020 was observed, regardless of groups, suggesting players were hypohydrated prior to the onset of exercise^17,39^. Given the data of pre-practice USG and body weight changes, players consumed an insufficient amount of fluid outside of and during practice. On the other hand, pre- and post-practice USG and urine electrolyte concentrations were lower in SCT players compared to CON. Even though the present study did not control for total fluid intake, it could be hypothesized that this result was associated with greater fluid consumed by SCT compared to CON. Recently, the NCAA guidelines required athletic departments to improve SCT players' education on the importance of hydration, heat-related illness prevention, and pre-season conditioning. Our athletic training staff, nutritionists, and coaches are instructed to monitor SCT players during conditioning and on-field practice constantly and verbally encourage them to consume fluids during the practice breaks rather than in response to thirst alone. In addition, our recent research on fluid balance and electrolyte losses in players who frequently complete in hot and humid environments demonstrated clear benefits to the dual consumption of carbohydrate-electrolyte solution and water during practice^17^. Further, a greater BUN/Creatinine ratio was also observed in CON at post-camp, which suggests CON players were experiencing chronic dehydration during the training camp periods. Therefore, although CON players do not appear at risk for negative health outcomes during competitions and training, the importance of fluid balance and heat-related illness still needs to be reinforced to avoid heat injuries in either group. Furthermore, higher sweat electrolyte and lower urine electrolyte concentrations were found in SCT players. This might suggest SCT players have compensatory sweat and urine mechanisms to normalize total electrolyte losses with training. Given the losses of electrolytes in SCT are likely higher sweat, a recommendation could be made to consume more sodium-rich beverages, as we have recommended in the past^17^. Future research needs to be conducted to better understand the mechanisms of electrolyte balance in SCT, possibly in controlled, laboratory settings.

A major strength of this study is that it is the first known that addresses the alterations of hematological and hydration status in elite collegiate football players with SCT in a longitudinal study design compared to position-matched controls. A previous study suggested that much of the published information on SCT and exertion injuries consisted of older data or case studies^40^. The present study provides valuable new information for the NCAA to develop additional guidelines to reduce the possibility of exertion-related injuries during conditioning or on-field practice in SCT athletes by demonstrating significant differences that exist in SCT compared to CON responses to training and competition. In addition, we outlined differences in hematological variables during crucial time points across a season that will allow medical staff to monitor future athletes more accurately for “normal” and “abnormal” SCT responses. A limitation to this study is that fluid intake was not measured during the camp training practices, and we cannot conclude that lower urine electrolytes were associated with more fluid intake or other factors. In addition, three SCT players were recruited in both years, which could be a potential confounding variable that prevents us from knowing the true variability in response in SCT. However, three new CON players were paired with the existing SCT players in year 2 to ensure adequate technical control and data validity. Last, this is an observational, field-based study, thereby increasing the response variability due to a lack of control of confounding variables and, to some extent, when assessments were scheduled. However, while this is a limitation, it is also a relative strength in that the results of this study can be directly generalized to those in active sport.

## Conclusions

In the present study, blood biomarkers showed that SCT players exhibited 31–40% HbS of hemoglobin and greater red blood cell distribution width compared to CON. In addition, serum uric acid remained greater in SCT players throughout the entire season. SCT players also had an improved fluid balance compared to CON but had lower urine electrolyte concentrations and greater sweat electrolyte concentrations during training camp. Given the results of the present study, it is of paramount importance to continue educating SCT players on the importance of fluid balance to avoid dehydration and heat exertional injuries. In addition, long-term conditioning and on-field training seems do not appear to affect the immune function in SCT players, however; the differences of uric acid and CPK levels showed SCT players might have a degree of physiological alterations in kidney function and muscle damage. The findings of the present study can significantly add to the screening process and extend the NCAA guidelines for SCT student-athletes. In addition, athletic training staff and physicians can better monitor the blood biomarkers in SCT players during exhaustive training and competitions via repeated blood testing and knowledge of ‘normal” seasonal changes in blood biomarkers in SCT. Future research should explore laboratory-based alterations of hematological biomarkers in SCT players during exhaustive exercise under hot and humid environmental conditions to further verify the results of the present study.
